# Nonsurgical Management of a Large Periapical Lesion: A Case Report 

**DOI:** 10.22037/iej.2017.49

**Published:** 2017

**Authors:** Sajedeh Ghorbanzadeh, Hengameh Ashraf, Sepanta Hosseinpour, Fatemeh Ghorbanzadeh

**Affiliations:** a * Department of Endodontics, Dental School, Shahid Beheshti University of Medical Sciences, Tehran, Iran;*; b *Research Fellow, Dental Research Center, Institute of Dental Sciences, Students' Research Committee, Shahid Beheshti University of Medical Sciences, Tehran, Iran;*; c * Dental School, Birjand University of Medical Sciences, Birjand, Iran*

**Keywords:** Mandibular Incisor, Nonsurgical Endodontic Therapy, Radicular Cyst

## Abstract

This case report describes the non-surgical management of a large cyst-like periapical lesion in the mandible of a 16-year-old female with the chief complaint of periodic swelling and pus drainage from the mandibular anterior region gingivae with no history of pain and traumatic accident in this area. Both mandibular central incisors had extensive caries. Root canals of both mandibular central incisors were filled with calcium hydroxide. After 10 days, endodontic therapy was carried out on both teeth. Clinical and radiographic re-evaluations at 3 and 12 months revealed progressing bone healing. This case report shows that appropriate diagnosis in combination with root canal treatment as a conservative non-surgical approach can lead to complete healing of large lesions without invasive treatments.

## Introduction

Pulpal tissue infection can occur as a result of many factors like caries or trauma which causes tissue necrosis. Periapical tissue eradication develops in response to microbial assumption and their by-products that infiltrate the periradicular tissues and activate the host's immune reaction [1]. Dynamic encounter between the host's immune response and microbial infective factors at the interface of the periodontal membrane and infected pulpal tissue results in various periapical lesions [2, 3]. In spite of the defensive and preventive nature of these periradicular lesions to microbial infection, they are not self-healing [4]. Incidences of radicular cysts in these lesions have been reported to be in the range of 6 to 55%. Moreover, the prevalence of periapical granuloma varies from 9.3 to 87.1%, and periapical abscess from 28.7 to 70.7% [1, 5]. It seems that when the radiographic size of the lesion becomes larger than 200 mm^2^, the occurrence of the cysts is more than 92% [6, 7]. 

Treatment approaches to handle large periapical lesions range from non-surgical endodontic therapy with or without endodontic surgery to tooth extraction. Microbial elimination or minimization from the pulp system using efficient chemo mechanical preparation can lead to a successful treatment [8]. Previous investigations demonstrated that large periapical lesions may be treated by nonsurgical endodontic approaches [9-11]. In fact, this usually occurs when the lesion has direct communication with the root canal system which can be improved with pus drainage through access cavity preparation [12]. On the other hand, when the lesion is separated from the apical foramen and covered thoroughly by an intact epithelium, it may not heal after non-surgical therapies [1, 7]. 

Appropriate anatomical knowledge and attention to preoperative radiography are also important in achieving desirable outcomes. Mandibular incisors in over 45% of cases present second canals [13-15] and their endodontic therapy may fail due to missed root canals. The following case report describes a nonsurgical management of a large mandibular cyst-like periapical lesion involving central incisors.

**Figure 1:**
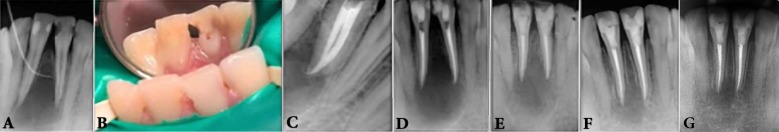
*A)* Periapical view showing a part of gutta-percha for tracing; *B)* Pus discharge after access cavity preparation; *C)* Intra canal paste dressing; *D)* Periapical radiograph after root canal filling; *E)* Periapical radiograph demonstrating progressive bone healing after 3 months; *F)* after 12 months; *G)* After 18 months

## Case Report

A 16-year-old female attended to the Department of Endodontics at Shahid Beheshti University of Medical Sciences, Tehran, Iran, with the chief complaint of periodic swelling and pus drainage from mandibular anterior region. She reported no pain and traumatic accident in this area and her past medical history was not contributory. Extra-oral examination showed non-palpable lymph nodes and no facial swelling. Intra-oral examination showed a sinus tract in the alveolar mucosa adjacent to tooth #25. Tooth decay in proximal surfaces of tooth #24 were seen and periodontal pocket was absent. Thermal and electrical vitality tests were performed for all mandibular incisors including #24 and 25 which did not elicit responses in latter teeth. Also, grade I mobility was identified in tooth #25 with tenderness on palpation in adjacent mucosa. All anterior mandibular teeth were painless on percussion testing. A periapical radiography indicated a large unilocular well-defined radiolucency surrounding mandibular incisors with a single tracing gutta-percha (Figure 1A). Also, radiography showed diversion of central incisors roots. It was decided to perform root canal therapy on teeth #24 and 25. The tooth was anesthetized with 68 mg articaine 3% (Darou Pakhsh, Tehran, Iran) containing 0.017 mg epinephrine (1:80000) and isolated with rubber dam to prepare the access cavity with a round diamond bur (Dentsply, Maillefer, Ballaigues, Switzerland). After access cavity preparation and pulpal exposure on tooth #25, a suppurative yellowish fluid was drained through the cavity (Figure 1B). After discontinuation of the drainage, working length was determined, both lingual and buccal canals were prepared with crown down technique by #15-40 Flexo-File (Dentsply, Maillefer, Switzerland), followed by gentle irrigation with sodium hypochlorite (2.5% NaOCl) and normal saline. Subsequently, root canals were filled with calcium hydroxide powder (Merck, Darmstadt, Germany) in combination with anesthetic solution as a paste using a lentulo ﬁller. The access cavities were sealed using 3 mm Cavit (ESPE, Seefeld, Germany). After 10 days, the dressing paste was irrigated by flushing with normal saline and teeth were asymptomatic. Root canals were repeatedly debrided and lateral condensation technique was used to obturate the canals with gutta-percha and AH-26 sealer (De Trey, Konstanz, Germany) (Figures 1C and 1D). The 3-, 12- and 18-month follow-ups indicated no sensitivity to palpation and percussion in clinical evaluations and radiographies showed evidence of developing bone regeneration (Figures 1E to 1G).

## Discussion

Inflammatory periradicular lesions which originated from endodontic conditions commonly range between 5 to 8 mm in diameter [16, 17]. Historically, periradicular lesions of up to 10 mm in diameter are considered as periapical granulomas and larger ones were recognized as periapical cysts [18, 19]. Epithelial cell rests of Malassez in the periodontal membrane are responsible for creating the epithelial lining of cysts. Radicular cysts may grow *via* a diversity of mechanisms, such as proliferation of epithelial cells or osmotic intensification [20]. The present case exhibited a large periapical lesion, which according to the above mentioned criteria, was most likely a periapical cyst. The existence of straw-colored exudate, size of lesion in combination with two non-vital teeth, radiopaque border, and divergence of adjacent teeth roots [21, 22] strongly demonstrated a radicular cyst. However, a definitive diagnosis can only be made using histopathologic examinations.

Various treatment options presented for large periradicular lesions may range from a root canal therapy to different surgical interventions [1, 23]. Sufficient chemo mechanical cleaning of the root canal system and appropriate microbial removal are the most essential factors for achieving satisfactory outcome. Previous studies have indicated that nonsurgical RCT should be done at first [8] which according to reports have shown that 42 to 74% of these lesions healed after RCT [11, 24, 25]. However, there is controversy about the differences between prognosis of conventional RCT of large and small lesions [11, 26]. 

An antibacterial calcium hydroxide-based paste dressing was placed in this case. The whole mechanism of action for this paste is still unclear. It is recommended that calcium hydroxide paste can improve periapical repair and eliminate residual microorganisms *via* handling the inflammation, stimulation of calcification, nullify acidic products of osteoclasts, and endotoxin neutralization [27-30]. Moreover, it has been shown that calcium hydroxide dressing strongly promotes periapical healing, notably in young adults [31, 32]. In agreement with these studies, periapical bone healing occurred 3 months after endodontic treatment in this case and continued over the next 9 months. Radiographic evaluations demonstrated bone regeneration according to increasing density, trabecular reconstruction, and lamina dura forming. 

Permanent restoration after endodontic treatment affects the prognosis and sufficient coronal restoration should be placed as soon as possible [33, 34]. In this case immediately after obturation, composite restorations on both teeth were placed. 

The number of visits for RCT is also one of the most controversial topics in endodontics [35, 36]. This case as a two-visit treatment confirms that two-visit RCT can result in successful healing. 

The benefits of less invasive nonsurgical treatment of extensive periapical lesions includes minimum psychological trauma and is more acceptable for patients. It seems that the periapical lesion was completely resolved due to rich blood supply, ample undifferentiated cells, and drainage through the lymphatic system. Thus, the lesion was rehabilitated in order to eliminate the causative factors by endodontic therapy and healing potential of periradicular tissues.

## Conclusion

In the present case, an extensive periradicular lesion with the clinical and radiographic aspects of a radicular cyst was treated with calcium hydroxide intracanal paste and root canal therapy. This confirms that large inflammatory periapical lesions can heal approvingly by nonsurgical therapy.
